# The Power of Images for Prognosis: A Rare Inferior Vena Cava Mass in a Patient With Atypical Symptoms of Heart Failure

**DOI:** 10.7759/cureus.69411

**Published:** 2024-09-14

**Authors:** Regina McPherson, Marina Shehata, Guillermo Izquierdo-Pretel

**Affiliations:** 1 Internal Medicine, Florida International University, Herbert Wertheim College of Medicine, Miami, USA

**Keywords:** inferior vena cava syndrome, ivc thrombus, ivc tumor invasion, renal cell carcinoma, renal mass

## Abstract

Inferior vena cava (IVC) syndrome is a cluster of symptoms that occur due to obstruction or stenosis of the inferior vena cava. Symptoms include lower extremity swelling and erythema, shortness of breath, and abdominal discomfort. This case describes a patient who presented for evaluation of lower extremity swelling and weakness associated with nausea and vomiting. A plan was made to investigate a cardiopulmonary etiology; however, the initial bedside ultrasound that included the evaluation of the abdomen discovered a mass in the IVC and left kidney. Additional imaging and intervention that involved a posterior radical nephrectomy and IVC thrombectomy allowed confirmation of left renal cell carcinoma (RCC) extended into the IVC with a thrombus causing the constellation of symptoms. A detailed history is always important to configure the IVC syndrome, but a quick and easy bedside ultrasound can identify IVC and other abdominal pathologies. A multidisciplinary approach is highly recommended for optimal results.

## Introduction

Renal cell carcinoma (RCC) stands as one of the most prevalent types of kidney cancer among adults. It is a neoplasm that originates from renal tubule cells within the renal cortex. The disease can be classified based on three histological subtypes: clear cell, papillary, and chromophobe, with clear cell being responsible for over 75% of cases [1]. Data shows that patients with clear cell RCC are diagnosed at a more advanced stage, and thus disease-specific survival is worst in this population. Risk factors for RCC include hypertension, obesity, acquired cystic kidney disease, and tobacco use. Exposure to trichloroethylene has been shown to lead to RCC development and increased mortality. Additionally, Von Hippel-Lindau is a familial disease noted to be associated with the development of clear cell RCC. RCC often progresses silently without manifesting significant symptoms in its early stages, making its detection challenging until it reaches advanced stages. Approximately 50% of patients are asymptomatic until the disease becomes advanced; hematuria, flank pain, and abdominal mass are common presentations in advanced disease. Symptomatic patients can present with bilateral lower extremity edema due to tumor invasion into the inferior vena cava. About 20% of patients present with symptoms of paraneoplastic disease: hypercalcemia, polycythemia, and hypertension [2]. However, the clinical course varies with RCC. The extent of the disease, the histological data, and the staging/grading of the tumor are needed to determine the prognosis of RCC [3]. The management and therapeutic options of RCC are dependent on staging, with nephrectomy or partial nephrectomy being common with tumors confined to the kidney [4]. 

Inferior vena cava (IVC) syndrome is a distinct clinical entity that is characterized by the invasion or compression of the IVC. This syndrome can present as a constellation of symptoms ranging from lower extremity erythema, swelling, and pain with exertion and abdominal discomfort to more severe complications such as renal dysfunction and venous thromboembolism. Patients may also experience dyspnea on exertion and low back pain [5]. These symptoms are usually a result of a reduced venous return to the heart and blood pooling in the IVC [6]. Despite the distinct nature of RCC and IVC syndrome, they ultimately intertwine, with RCC serving as one of the primary causes of IVC syndrome. If there is tumor invasion into the IVC, segmental resection and IVC reconstruction are generally necessary and typically the only curative approach [7]. It is not uncommon for IVC syndrome to be misdiagnosed as it may mimic other disease processes. A high index of suspicion is required to make the diagnosis. Therefore, understanding the clinical manifestations and management strategies of IVC syndrome and its association with RCC is crucial for healthcare professionals to provide comprehensive care to affected individuals.

We present a case of a 49-year-old female who presented with symptoms of IVC syndrome and was found to have advanced renal cell carcinoma. 

## Case presentation

The patient is a 49-year-old female who presented to the emergency room with complaints of nausea, vomiting, lower extremity swelling, and weakness for three days. Symptoms occurred suddenly with the inability to climb stairs with pain in the lower extremities. The patient noted erythema of the bilateral lower extremities with walking. Subsequently, she developed severe dizziness and had an episode of emesis, after which she presented to the emergency room. Before presenting to our service, she was admitted to a peripheral facility, where she was attended for possible cardiac issues. The patient denied any episodes of hematuria or back pain. She reported a past medical history of type 2 diabetes and hypertension controlled with oral medications. The patient reported an extensive family history of cancer, which included breast cancer in her mother, colon cancer in her grandfather, ovarian cancer in her grandmother, and lung cancer in her uncle. Past surgical history was significant for a hysterectomy due to uterine leiomyoma and cesarean section. The patient denied any history of tobacco, alcohol, or illicit drug use and no significant occupational exposures. 

**Figure 1:**
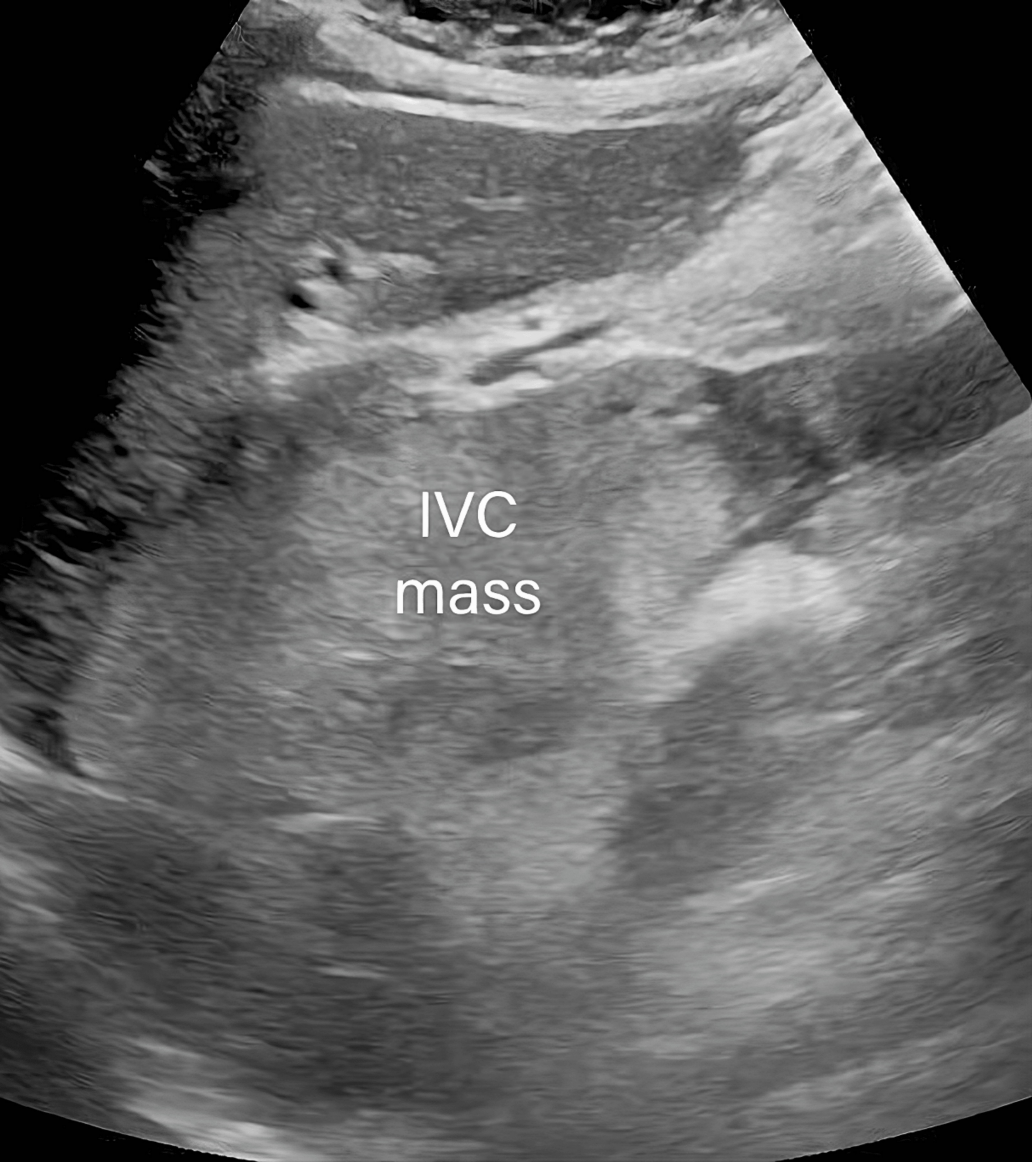
Heterogeneous mass within the IVC IVC: inferior vena cava

**Figure 2:**
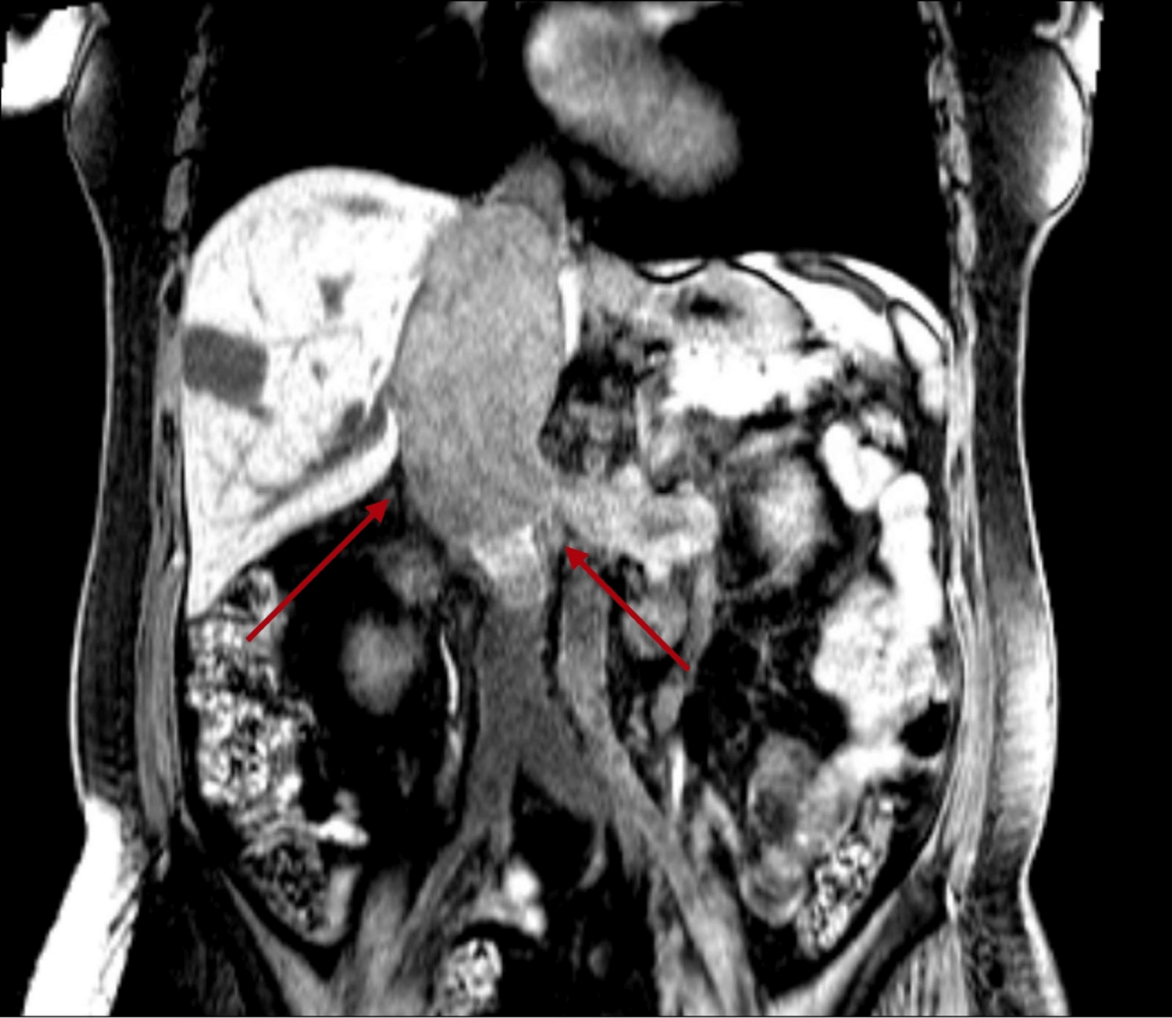
Tumoral thrombosis from the left renal vein extending to the supradiaphragmatic IVC and to the infrarenal IVC spanning approximately 12.1 cm IVC: inferior vena cava

On examination, the abdominal exam was notable for epigastric tenderness to deep palpation but no costovertebral tenderness. Lower extremities had trace pitting edema up to the ankles bilaterally. The remaining physical exam was unremarkable. Initial laboratory results were significant for elevated liver function enzymes; other laboratory results were unremarkable (Table 1). Abdominal ultrasound described multiple masses within the abdomen, including a 5.1 x 10.3 x 3.8 cm heterogeneous mass within the IVC (Figure 1), a 2.9 x 2.9 x 3.2 cm mass within the right hepatic lobe, and a 6 x 5.3 x 3.9 cm mass within the superior pole of the left kidney. A 2D echo showed normal cardiac function. Magnetic resonance angiography of the abdomen with and without contrast was significant for a combination of tumoral thrombosis and bland thrombus in the IVC (Figure 2). Tumoral thrombosis from the left renal vein was extending to the supradiaphragmatic IVC and the infrarenal IVC, spanning approximately 12.1 cm. Immediately inferior to the tumor thrombus, there is a bland thrombus in the infrarenal IVC spanning 3.2 cm. A 3.1 cm hepatic lesion T1hypo/T2hyper-intense with progressive peripheral nodular enhancement in segment 6/7 and an additional 1.2 cm similar lesion in segment 7 favored hepatic hemangioma. 

**Table 1:** Laboratory results on admission BUN: blood urea nitrogen; CPK: creatine phosphokinase; AST: aspartate transaminase; ALT: alanine aminotransferase; BNP: B-type natriuretic peptide

Lab value	Results	Reference ranges
BUN	15 mg/dL	7 mg/dL – 17 mg/dL
Creatinine	0.60 mg/dL	0.52 mg/dL – 1.04 mg/dL
CPK	26 units/L	35 units/L - 230 units/L
AST	268 units/L	15 units/L - 46 units/L
ALT	674 units/L	9 units/L – 30 units/L
Pro-BNP	82.7 pg/ml	0 pg/ml– 125.0 pg/ml
Troponin	<0.012 ng/ml	0.000 ng/ml – 0.034 ng/ml

Initial management was conservative, which included pain management. Oncology was consulted and recommended a multidisciplinary approach to the case. After discussion with urology and presentation at the tumor board, the patient underwent left nephrectomy, left IVC thrombectomy, IVC filter placement, and primary repair of the IVC. The patient developed acute posthemorrhagic anemia and required two units of packed red blood cells (pRBCs). Renal function remained stable with no need for renal replacement therapy. The patient was transferred to the coronary care unit (CCU) for continued management and evaluation.

The pathology report showed clear cell renal cell carcinoma in the left kidney with invasion to the renal vein and the presence of carcinoma in the resected inferior vena cava with associated thrombus. The patient was seen by outpatient oncology one week after hospital discharge with plans to start adjuvant immunotherapy with pembrolizumab for one year. The patient requested to pursue further treatment closer to her home. Appropriate oncology referrals were sent. 

## Discussion

Renal cell carcinoma is the most common kidney cancer in adult patients. Patients with RCC may initially be asymptomatic when the tumor is small, and the mass is discovered incidentally in routine studies. As the mass progresses in size, patients may develop hematuria, flank pain, fatigue, weight loss, back pain, anemia, and high serum calcium levels. The well-known classic presentation of RCC hematuria, flank pain, and flank mass is uncommon and only present in about 10% of patients [8]. Of note, this presentation typically indicates an advanced disease. Surgical resection is the treatment choice for RCC. IVC syndrome is a possible presenting feature of RCC when the tumor invades the IVC or due to tumor thrombosis. IVC invasion is a negative prognostic factor, and surgical resection is the only curative option [9]. While IVC syndrome is common in patients with congenital anomalies and often presents because of thrombotic complications, it is still a rare complication. RCC can cause an acquired anomaly in the IVC and thus the development of IVC syndrome in these patients [10]. A high index of suspicion is needed to make the diagnosis, as the presenting symptoms can mimic a wide array of other conditions, so misdiagnoses are common. Patients can present with back pain, lower extremity swelling, and discomfort in the legs; these features can be in the absence of the common presenting symptoms seen with RCC. 

When RCC with IVC invasion by the tumor is suspected, imaging studies should be done to assess the extent of IVC wall invasion. IVC wall invasion by the tumor presents a challenge, and depending on the extent of invasion, it may require IVC resection. Typically, treatment of IVC syndrome is essentially focused on eradication of the primary disease via chemotherapy; however, vascular bypass and resection of the tumor can be indicated [11]. In patients with RCC and IVC invasion, studies suggest that the five-year survival rate was only 26% in patients who did not undergo resection and/or thrombectomy of the invaded IVC wall. Percentages reached as high as 57% in patients who proceeded with resection [12]. Therefore, it is necessary to resect the invaded IVC vessel wall. In this case, the patient presented with non-specific symptoms initially thought to be cardiac/pulmonary related, and her initial workup was aimed at identifying a cardiopulmonary pathology. A quick bedside ultrasound was able to identify a mass in the IVC. Subsequent imaging identified multiple masses within the abdomen, including within the IVC and the superior pole of the left kidney. This case is distinct as the patient presented with atypical symptoms of heart failure, for which an initial investigation with ultrasound identified an IVC mass. The case contributes to the existing literature, providing emphasis on creating a wide differential and consideration of acquired anomalies in the IVC such as that caused by RCC when patients present with these rare features. It also serves to highlight the use of ultrasound as a rapid and reliable initial tool to evaluate patients at the bedside.

## Conclusions

IVC syndrome is a rare condition that clinicians should identify with a detailed history and physical examination. As there are no pathognomonic symptoms for IVC syndrome, a bedside point-of-care ultrasound (POCUS) involving the IVC evaluation can help identify thrombi. Additional workup should be completed to identify the underlying cause and the extent of the obstruction. Due to anatomical implications, RCC can lead to IVC syndrome through direct tumor invasion within the IVC. IVC syndrome secondary to RCC represents a complex clinical scenario requiring a multidisciplinary approach for optimal patient outcomes.
